# Encapsulating Non-Human Primate Multipotent Stromal Cells in Alginate via High Voltage for Cell-Based Therapies and Cryopreservation

**DOI:** 10.1371/journal.pone.0107911

**Published:** 2014-09-26

**Authors:** Oleksandr Gryshkov, Denys Pogozhykh, Nicola Hofmann, Olena Pogozhykh, Thomas Mueller, Birgit Glasmacher

**Affiliations:** 1 Institute for Multiphase Processes, Leibniz Universitaet Hannover, Hannover, Germany; 2 Institute for Transfusion Medicine, Hannover Medical School, Hannover, Germany; University of Torino, Italy

## Abstract

Alginate cell-based therapy requires further development focused on clinical application. To assess engraftment, risk of mutations and therapeutic benefit studies should be performed in an appropriate non-human primate model, such as the common marmoset (*Callithrix jacchus*). In this work we encapsulated amnion derived multipotent stromal cells (MSCs) from *Callithrix jacchus* in defined size alginate beads using a high voltage technique. Our results indicate that i) alginate-cell mixing procedure and cell concentration do not affect the diameter of alginate beads, ii) encapsulation of high cell numbers (up to 10×10^6^ cells/ml) can be performed in alginate beads utilizing high voltage and iii) high voltage (15–30 kV) does not alter the viability, proliferation and differentiation capacity of MSCs post-encapsulation compared with alginate encapsulated cells produced by the traditional air-flow method. The consistent results were obtained over the period of 7 days of encapsulated MSCs culture and after cryopreservation utilizing a slow cooling procedure (1 K/min). The results of this work show that high voltage encapsulation can further be maximized to develop cell-based therapies with alginate beads in a non-human primate model towards human application.

## Introduction

**Figure 1:**
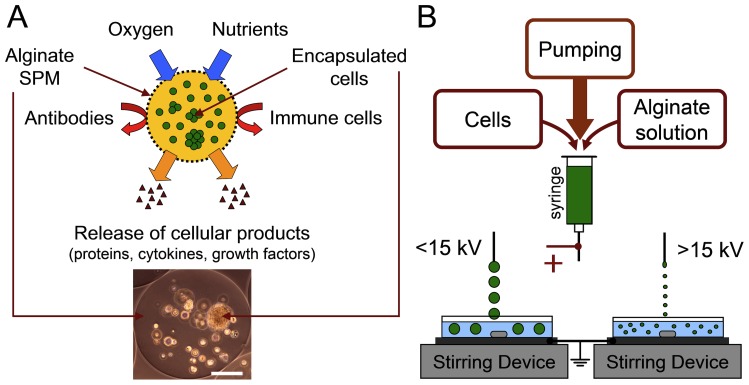
Schematic presentation of alginate high voltage encapsulation. (**A**) Application of encapsulation of cells in alginate using high voltage (**B**) in cell-based therapy for immunoisolation, controllable drug release through semi-permeable membrane (SPM) and long-term storage of cells. Scale bar is 100 µm.

Cell-based therapies are under development to treat a wide range of acute and chronic diseases. To date, they have been successfully applied in treatments of the central and peripheral nervous system [1], bone and cartilage regeneration, hepatic fibrosis and cardiac insufficiencies [2], [3]. The main challenge in such allogenic therapies is the suppression of the host immune system prior to and during the treatment. In addition, drug-based immune system suppression has many side effects for the patient [4]. One strategy to avoid harmful immunosupression of the host is the suppression of the major histocompatibility complex I (MHC I), a major obstacle in transplantation, in the transplanted cells by small hairpin RNA (shRNA) method [5]. Alternatively, cells can be encapsulated into polymer matrices with semi-permeable properties; these shield transplanted cells from immune responses, while allowing controlled release of drugs and cellular products [6]. Interestingly, most matrices mimic the extra-cellular matrix and therefore provide the cells with a niche-like environment during post-transplantation (Figure 1A).

Alginate is known to be a linear block co-polymer containing sequences of (1–4)-linked β-D-mannuronate (M-residue), its C-5 epimer α-L-guluronate (G-residue) and alternating M and G residues (MG-residues). It can be produced from brown algae and bacteria. However, alginate extracted from different sources has variable properties and alginate beads produced by a range of cross-linking methods display a wide range of final biological and physical properties, affecting the mechanical properties of a bead and cell response *in-vitro* and *in-vivo* [7]. The features of cross-linking include: ionic cross-linking with divalent cations, such as barium and calcium, that provides living cells with a niche-like environment and ease of gelation, forming so called egg-box structure [8]; whereas covalent cross-linking with polyethylene glycol induced by laser irradiation enhances the degradation rate facilitating a more homogeneous cross-linking [9]. Cell cross-linking utilizing arginine-glycine-aspartate (RGD) coupled alginate improves attachment of adherent mammalian cell types [10], whereas thermal gelation with thermo-responsible materials allows cross-linking at body temperature [11]. In summary, by varying the type and density of cross-linking, the physical and biological properties of the bead can be controlled and matched with the required application.

To date, three major methods are suitable for encapsulation of living cells in alginate beads: 1) electro-spraying (ES), utilizing a voltage in order to detach alginate beads from the nozzle, 2) air-flow (AF), allowing bead production using air motion under pressure, and 3) ink-jet fabrication, a method based on piezoelectric effects [12], [13]. However, encapsulation methods require further optimization in order to reduce bead size while avoiding ruptured structures. Smaller alginate beads are preferable in cell culture and transplantation due to increased surface-to-volume ratio and enhanced heat and mass transfer to encapsulated cells. Here, the application of poly-cationic coatings, such as poly-L-lysine, has been shown to stabilize the structure of alginate beads and control molecular weight cut-off [14], but bead biocompatibility was significantly lower when compared to uncoated and unmodified calcium and barium beads [15], [16].

The terminology “mesenchymal stem cells” is misleading because the mesenchyme is embryonic connective tissue from mesodermal origin, differentiating in hematopoietic and connective tissue, which this cell type cannot. Therefore, “multipotent stromal cells” (MSC) is a far more accurate term for this interesting cell population which will be tested prior to clinical application in the common marmoset monkey (*Callithrix jacchus)* as a relevant preclinical non-human primate model. For future application in regenerative medicine, the introduction of such a model is more important than widely used rodent models due to high phylogenetic similarity of a marmoset to a human and derivation of embryonic (ESC), induced pluripotent (iPS) and adult stem cells [17]–[20]. In our experiments, MSCs were derived from the placental amnion membrane of the animals, offering a non-invasive strategy for retrieval and theoretical availability for each (future) patient. This is due to the fact that the amnion membrane is generated from the embryonal epiblast, whereas the chorion is originated from the trophoblast and the decidua from maternal origin [21]. Immediate availability of these cells can be assured by their long-term storage at low temperatures with appropriate cryopreservation procedures. This is currently the only possible technique for the long term storage of rare cell types. The preservation of stem cells with high viability, proliferation and yet preserving their differentiation potential called “stemness” still poses challenges. One strategy to improve viability and proliferation after cryopreservation deals with the encapsulation of cells in small-sized alginate beads before freezing. The gel-like structure, mild environment inside alginate beads and improved heat and mass transfer due to increased surface-to-volume ratio may protect encapsulated cells from cryo-injury and resist the reorganization of ice crystals during thawing.

Therefore, in this work, we applied high voltage ES to encapsulate MSCs in small alginate beads with defined diameter. We investigated the efficiency of the ES method to encapsulate high cell numbers and the effects of cell concentration and cell-mixing procedure on bead diameter. Furthermore, we evaluated the effect of high voltage encapsulation procedure and cryopreservation on viability, proliferation and differentiation of MSCs after encapsulation, using the AF method as a comparative approach.

## Materials and Methods

All chemicals used in the study were purchased from Sigma, unless indicated otherwise.

### Alginate: source and sterilization

Medium viscosity alginate sodium salt from brown algae (molecular weight 80000–120000 g/mol, viscosity 2000 cP (2%, 25°C)) was dissolved in 10 mM 4-(2-hydroxyethyl)-1-piperazineethanesulfonic acid (HEPES, Carl Roth GmbH, Karlsruhe, Germany) buffered saline (pH 7.4) at a concentration of 1.6% (w/v) with gentle shaking overnight. Aliquots of alginate solution were kept at 4°C until use. Raw alginate solution was sterilized using 0.8-0.45-0.2 µm filters. Membrane filtration of alginate solution was used to sterilize the product, as this the only type of sterilization that does not cause significant changes in molecular weight and viscosity of alginate [22]. Sterile alginate solution was transferred to 10 ml sterile syringes and kept at 4°C. Aliquots of alginate solution were left to warm up at room temperature (RT) one day prior to the experiments.

### Encapsulation methods

We have previously designed the high voltage ES encapsulation method and optimized the process parameters. It applies a high voltage to overcome the surface tension of the alginate solution (Figure 1B). Briefly, the alginate solution is held in a syringe and passed through an adaptor under controlled flow rate to the nozzle (blunt needle, outer/inner diameters 0.4/0.2 mm, length 25 mm, BBraun, Melsungen, Germany). Alginate gel is detached from the tip of the nozzle by applying a high voltage between the needle and the grounded electrode with following cross-linking in the bath containing stirred solution with Ca^2+^ ions. Calcium ions replace sodium ions in alginate due to their higher affinity to the alginate, forming a gel-like structure. We compared our approach to the commonly used AF method that does not apply a high voltage to generate alginate beads. The detailed description of the AF encapsulation method has been published previously [12]. In brief, a high velocity air flow overcomes the surface tension of the alginate solution resulting in bead detachment. The AF method has previously been optimized for the production of alginate beads with a narrow size distribution compared to the beads obtained using ES approach (bead diameter 300–400 µm) [23].

The size of generated alginate beads was determined to investigate the encapsulation efficiency. Briefly, the alginate beads (with or without encapsulated cells) were cross-linked after encapsulation for 30 min (unless stated otherwise). After cross-linking, the diameter of alginate beads generated by the ES or AF method was measured using an Axiovert 200M microscope (Carl Zeiss, Germany). Pictures were taken in bright-field at 5X magnification and analyzed using AxioVision software (Rel. 4.7) (8–10 images, 10–15 beads per image). The average bead diameter of one hundred beads was determined.

### Cell derivation, characterization, culture and encapsulation

#### Ethics statement

No animal experiments were performed in this study. Donation of the placenta material after natural birth’s was carried out from the Center for Reproductive Medicine and Andrology Muenster (CeRA) by institutional animal care takers and approved by the Institutional Animal Care. The ethical husbandry of *Callithrix jacchus* in this institute was in accordance with the guidelines of the Ministry for Environment and Nature Protection, Agriculture and Consumer Protection (LANUV), North-Rhine Westphalia, Germany.

#### Cell derivation and isolation

Placental samples were collected after natural birth from healthy marmosets in the CeRA. Placentas were washed with phosphate buffered saline (PBS, Biochrom AG, Germany) with 1% Ciprobay (Fresenius Kabi, Bad Homburg, Germany). Then the amnion membrane of each part of placenta was removed and dissected into smaller pieces. The amnion membrane pieces were incubated with 0.25% trypsin for 1 h at 37°C, filtered through 100 µm cell strainers and centrifuged at 1,200 rpm for 5 min. The cell pellet was re-suspended in MSC growth medium and plated into 10 cm cell dishes. All cell samples were tested for mycoplasma contamination.

#### Cell culture and encapsulation

MSCs derived from the common marmoset monkey *Callithrix jacchus* were cultured under sterile conditions in Dulbecco’s modified Eagle’s medium (DMEM, Biochrom AG, Germany) containing 15% (v/v) fetal bovine serum (FBS, Biochrom AG, Germany), 1% penicillin/streptomycin, 1% ascorbic acid in 10 cm tissue culture dishes (TPP, Biochrom AG, Germany) in a humidified incubator at 37°C and 5% CO_2_. The cells were harvested using 0.05%/0.02% (w/v) trypsin/EDTA solution followed by centrifugation at 1,200 rpm for 5 min. Membrane integrity of MSCs was assessed by trypan blue staining using Vi-CELL XR cell viability analyzer (Beckman Coulter GmbH, Krefeld, Germany). After counting, MSCs were centrifuged and re-suspended in 10 mM HEPES buffered saline (pH 7.4) to adjust the desired cell concentration before encapsulation in alginate. The encapsulation was performed by the AF and ES method; here, the cells were gently mixed with 1.6% alginate in 10 ml syringe and pumped at 10 ml/h flow rate into 100 mM CaCl_2_ cross-linking solution (in 10 mM HEPES buffered saline). The obtained alginate beads containing MSCs were washed twice with washing solution (WS), containing 10 mM HEPES, 1.5 mM CaCl_2_ buffered saline (pH 7.4), and either immediately used for experimental purposes after encapsulation (“Immediate”) or after a day of culture in a humidified incubator at 37°C and 5% CO_2_ (“Incubated”). The presence of calcium ions in WS prevented alginate beads from swelling [24]. The following process parameters were kept constant during encapsulation using both the AF and ES method: spraying distance 10 cm, alginate flow rate 10 ml/h, concentration of CaCl_2_ solution 100 mM, applied voltage 20 kV, air flow 150 l/h, unless stated otherwise.

#### Flow cytometry marker analysis

After trypsinization and fixation in 4% paraformaldehyde (PFA, Merck, Germany), cells were aliquoted in equal numbers for flow cytometry (FACS Calibur, Becton Dickinson) and stained with primary and secondary antibodies at RT for 1 h, respectively (Table S1). Cells incubated with only secondary antibody were used as negative controls.

#### Immunohistochemical staining

For immunohistochemical staining Dako LSAB+System-HRP kit was used (Dako North America, California, USA). For the examination for mesenchymal markers, 2×10^4^ cells/well of each sample were seeded on a glass slide (20 mm) in a 12-well plate (Greiner Bio-One GmbH, Frickenhausen, Germany) and then fixed with 4% PFA. After washing steps with PBS, six drops of 3% hydrogen peroxide were added to each well for 5 min to block endogenous peroxidase activities. The cells were incubated with appropriate antibody over night at 4°C (Table S1). Then the link solution was applied to the glass slides for 30 min followed by streptavidin peroxidase for 30 min. The substrate solution was added to each well and incubated for 7–15 min until the antibody horseradish peroxidase conjugate (AB-HRP) catalyzed the 3,3′-diaminobenzidine (DAB) substrate into the desired brown to black reporter color for a positive signal. Afterwards, cells were washed twice with 1 ml distilled water (dH_2_O) and treated with 300 µl haematoxylin for 3–5 min providing counterstaining of the nuclei. The glass slides were additionally rinsed with dH_2_O, transferred upside down onto an object plate in one drop of Mowiol 4–88 (Carl Roth GmbH, Karlsruhe, Germany) and dried over night in the dark. Images were taken using a Keyence Biozero microscope (Keyence Germany GmbH, Neu-Isenburg, Germany).

#### Total RNA Isolation and Reverse Transcription Polymerase Chain Reaction (RT-PCR)

RNA was extracted using the peqGOLD Total RNA Kit (Peqlab GmbH, Erlangen, Germany) according to the manufacturer’s instructions. Briefly, the cell pellet was lysed in 400 µl RNA Lysis Buffer, transferred to a provided DNA removing column and centrifuged at 12,000 g for 1 min. Next, 400 µl 70% ethanol were added to the flow through, the lysate was loaded onto a provided Perfect Bind RNA Column and centrifuged at 10,000 g for 1 min. After three washing steps (1x 500 µl RNA Wash Buffer I and 2×600 µl RNA Wash Buffer II), the column was dried by centrifugation at 10,000 g for 2 min and the RNA eluted from the column by applying 50 µl sterile RNAse-free dH_2_O. Concentration of the obtained RNA was measured by a Nanodrop photometer (Spectrophotometer ND1000, Peqlab GmbH, Erlangen, Germany). Extracted RNA was transcribed into complementary DNA (cDNA) using the High Capacity cDNA Reverse Transcription Kit (Life technologies, Carlsbad, USA). By adding Oligo-17-dT primers (TIB Molbiol, Berlin, Germany) only the messenger RNA (mRNA) with intact poly-A tail was transcribed. For the analysis of the mesenchymal markers and immunorelevant molecules, a RT-PCR reaction of 30 µl per sample was set up with standard conditions (24 µl dH_2_O, 3 µl 1X PCR buffer (NEB, Frankfurt, Germany), 0.5 Units Taq Polymerase (NEB, Frankfurt, Germany), 100 mM nucleoside triphosphates containing deoxyribose (dNTPs, Fermentas, St. Leon-Rot, Germany), 20 pmol/µl of each primer and 1 µg cDNA). The RT-PCR reaction was performed in a Thermal cycler C1000 (Bio-Rad Laboratories GmbH, Munich, Germany). The cycling conditions were as follows: 1x initial denaturation step at 95°C for 180 s, followed by 35x denaturation at 95°C for 45 s, annealing at 58°C for 45 s, extension at 72°C for 90 s and a final extension step at 72°C for 600 s. In all amplifications, ribosomal protein S29 (RPS29) was utilized as a housekeeper gene; exact oligonucleotide sequences, corresponding fragment sizes and annealing temperatures are enlisted in Table S1.

### Optimization of cell number

Cell concentration (1×10^6^, 5×10^6^, 10×10^6^ cells/ml), alginate-cell mixing procedure and applied voltage (10, 15, 20, 25, 30 kV) was also optimized, since the incorporation of living cells may also change the viscosity and surface tension of final alginate-cell solution resulting in increased bead size, thus affecting the heat and mass transfer to the encapsulated cells. Concentration of alginate (1.6% in 10 mM HEPES buffered saline, w/v), cross-linking solution (100 mM in 10 mM HEPES buffered saline), spraying distance (10 cm), and alginate flow rate (10 ml/h) were kept constant. The diameter of alginate beads was measured as described above.

### Shrinking rate and encapsulation efficiency

Shrinking rate (SR) of alginate in presence of gelling agents (Ca^++^) was analyzed by comparing the initial volume of alginate droplets detached from the nozzle via gravity (manual dropping, MD), AF and high voltage. The volume of alginate beads was calculated by the diameter after cross-linking for 40 min. The volume at t = 0 revealed the initial volume of alginate droplet. The MD, AF and ES method was utilized to generate beads with different surface-to-volume ratios (S/V) as a result of initial bead diameter. The initial volume (also respective S/V ratio) was calculated by dropping the alginate solution on the collector and calculating the amount of generated droplets *vs.* dropping time. This preliminary experiment was performed ten times providing the resulted S/V ratios (assuming the alginate drop having a form of a sphere) of 2.47, 9.15, 8.28 and 11.47 mm^−1^ for 1.5%-MD, 1.2%-AF, 1.5%-AF and 1.5%-ES of experimental settings, respectively. The volume of alginate beads during gelling was calculated from measuring their diameter at defined time points during gelling: from 0 to 5 min with a step of 1 min, from 5 to 40 min with a step of 5 min. In general, SR can be presented as follows:
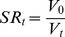
(1)whereas *V_0_* – initial volume of alginate drop, *V_t_* – volume of alginate bead at time t of cross-linking. The value of SR calculated for 1.5%-ES and 10 min of gelling was used to analyze the efficiency of encapsulation of MSCs in alginate at a concentration of 1×10^6^, 5×10^6^ and 10×10^6^ cells/ml. In this particular case (alginate concentration 1.5%, cross-linking time 10 min), assuming no release of cells from the alginate bead during spraying and gelling, the final number of cells encapsulated in alginate bead was evaluated as follows:
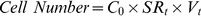
(2)whereas *C_0_* – initial cell concentration (cells/ml), *SR_t_* – shrinking rate of alginate after time t of gelling, *V_t_* – volume of alginate bead after time t of gelling (ml).

### Cell viability

MSCs were encapsulated into 1.6% alginate using AF and ES under high voltage (15, 20, 25, 30 kV) followed by washing steps. After washing, alginate beads were transferred to 6-well culture dishes (TPP, Biochrom AG, Germany) and cultured for 10 days in a humidified incubator at 37°C, 5% CO_2_ with daily cell culture medium change. At defined time points (day 0, 1, 3, 5, 7 and 10), approximately 20 beads were taken, washed twice with WS and stained for live-dead viability based on previously published protocol with some modifications [25]. CalceinAM indicated viable cells upon their enzymatic activity by green fluorescence (excitation/emission: 494/517 nm), whereas Ethidium Homodymer (CalceinAM/EthD, Sigma, USA) indicated dead cells with ruptured cell membranes in red (excitation/emission: 528/617 nm) by intercalating to the nuclear DNA of the cell nuclei. Equal volumes of beads containing cells in HEPES and working solution of the live-dead staining were mixed to get the final concentrations of 2 µM and 4 µM of CalceinAM and Ethidium Homodymer, respectively, and incubated for 10 min in the dark at RT. After staining beads were washed twice with WS and immediately imaged using an Axiovert 200M fluorescence microscope at 5× magnification to detect viable and dead MSCs. Separate images of live and dead cells were analyzed using AxioVision software (Rel. 4.7).

### Cell recovery

To evaluate the proliferation and differentiation capacity of differently encapsulated MSCs, the cells were allowed to release from the alginate beads by 55 mM sodium citrate in 10 mM HEPES buffered saline (pH 7.4) with gentle shaking for 5 min, thus replacing calcium ions by sodium ions. This resulted in formation of diluted sodium alginate and the release of MSCs from the beads. The cells were further collected by centrifugation at 1,200 rpm for 5 min and the proliferation and differentiation potential after encapsulation, incubation and cryopreservation evaluated. Because the recovery of cells might not be finished in only 24 h, we cultured the MSCs in 6-well culture flasks at a concentration of 1×10^5^ cells/well in a humidified incubator at 37°C and 5% CO_2_ for 5 days. After this period of time, we assessed the cell number, membrane integrity and further seeded cells for proliferation assay.

### Cell proliferation

The MSCs were seeded in 96-well culture flasks (TPP, Biochrom AG, Germany) at a concentration of 1×10^4^ cells/well for the CellTiter 96 Non-radioactive Cell Proliferation Assay (G4000, Promega, USA) following manufacturer’s protocol. Briefly, after 24 h in culture flasks in a humidified incubator (37°C/5% CO_2_), 15 µl dye solution of the proliferation assay was added to each well and incubated for 4 h to let living cells convert the tetrazolium component of the dye solution into formazan crystals. After the addition of 100 µl stop solution, the well was incubated for 1 h in the dark to solubilize the formed formazan crystals and the absorbance was measured at 570 nm wavelength using 96-well micro-plate reader (Multiscan Ex, Thermo Scientific, China). Absorbance values were normalized with controls of cultured cells without encapsulation and cryopreservation.

### Characterization of differentiation potential

MSCs were encapsulated using the high voltage (20 kV) and AF method, released from the beads, tested for viability as described above and seeded at a density of 5×10^4^ cells/well in 6-well culture dishes. The differentiation of MSCs towards adipogenic and osteogenic lineages was performed following the protocol published by our group [26].

#### Adipogenic

Adipogenic differentiation of MSCs was induced by culture of the cells for 5 days in medium containing 1 µM dexamethasone, 0.2 mM indomethacin, 0.5 mM 3-Isobutyl-1-methylxanthin and 10 µg/ml insulin in DMEM supplemented with 10% FBS. Detection of formed lipid vacuoles was assessed using Oil Red O stain as described earlier [26]. Briefly, the cells were washed with PBS, fixed in 4% PFA for 20 min and rinsed twice with tap water followed by one final wash with 50% ethanol (ROTH, Germany). Formed lipid vacuoles were detected by incubation for 10 min in Oil Red O in acetone/50% ethanol (Merck, Germany) and a final rinse with tap water. All differentiation procedures were repeated four times, compared with undifferentiated control cells (n = 4) and visualized with an Axiovert 200M microscope in bright field.

#### Osteogenic

The cells were cultured in osteogenic differentiation medium supplemented with 0.1 µM dexamethasone, 10 mM glycerophosphate, 0.05 mM L-ascorbic acid-2-phosphate and 1% ITS (BD Biosciences, Germany) in DMEM with 15% FBS. To detect mineralization of differentiated osteoblasts, Von Kossa staining was performed as described earlier [26]. Briefly, MSCs with the mineral deposit were washed twice in PBS and fixed with 10% PFA, washed once with PBS and twice with dH_2_O followed by addition of 1% silver nitrate (Ridel de Haen GmbH, Germany). In the following, the dish was exposed to sunlight for 30 min, washed with bi-distilled water (bi-dH_2_O), stained with 5% sodium thiosulfate for 5 min and again rinsed with bi-dH_2_O.

All differentiation procedures were repeated four times, compared with undifferentiated control cells (n = 4) and visualized with an Axiovert 200M microscope in bright field.

### Cryopreservation

The MSCs were encapsulated and cryopreserved at a concentration of 0.5×10^6^ cells/sample either immediately after encapsulation (“Immediate”) or after cultivation in culture medium in a humidified incubator at 37°C, 5% CO_2_ for 24 h (“Incubated”). Cells encapsulated in alginate under high voltage (15, 20, 25 kV) and using the AF method were washed twice with WS and exposed to 2X freezing medium at 4°C drop-wise (0.5 ml/min), containing 20% (v/v) dimethyl sulfoxide (DMSO, Carl Roth GmbH, Germany) and 20% FBS to obtain the final concentration of 10% DMSO and 17.5% FBS. Samples were equilibrated at 4°C for 15 min in 1.5 ml cryo-vials (TPP, Biochrom AG, Germany) and cooled down at 1 K/min (slow cooling protocol) down to −80°C with Mr. Frosty Freezing Container (Thermo Fisher Scientific, Schwerte, Germany), without active control over the temperature of extracellular ice formation (spontaneous ice formation). Frozen samples were transferred to −150°C and kept for 7 days before thawing at 37°C in a water bath for 2 min. The thawing rate was as follows: from −150°C to −25°C with 243 K/min, from −25°C to 4°C with 18 K/min (Figure S1). In order to reduce the negative effect of osmotic stress which takes place during fast dilution procedure, the thawed samples were further diluted by drop-wise addition of fresh culture medium at 4°C. The cells were released from the beads as described above, checked for membrane integrity by trypan blue staining and seeded for proliferation assay either immediately after thawing or after being recovered for 5 days in culture. As control, non-encapsulated MSCs in suspension were also cryopreserved under the same conditions.

### Data analysis and statistics

The data are shown as a mean±SD. Here, “n” represents the number of independent experiments with “N” samples each. Significance level was analyzed using one-way ANOVA with a mean comparison using Tukey method with Microsoft Excel 2007 software. The level of p<0.05 was assumed as significant.

## Results

### Characterization of the MSCs

**Figure 2:**
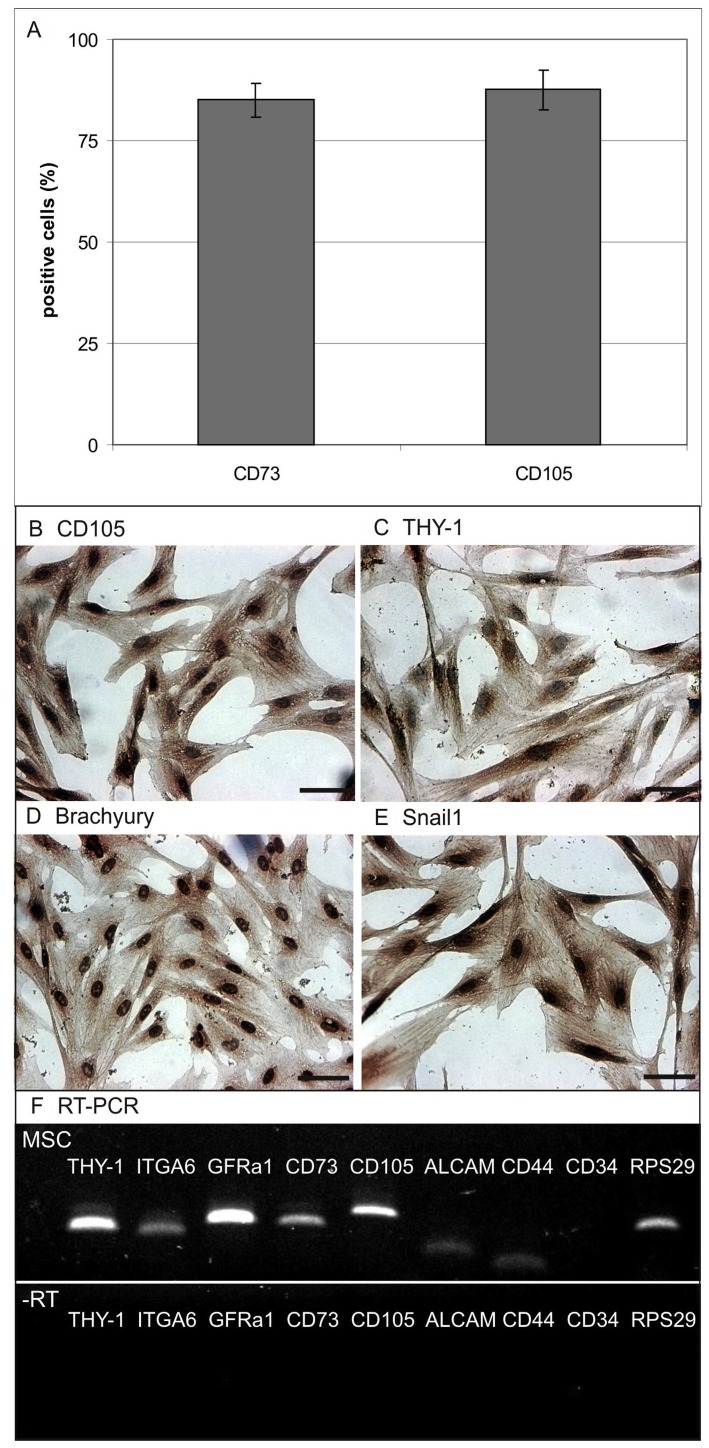
Characterization of amnion derived multipotent stromal cells. (**A**) FACS analysis with characteristic markers CD105 and 73 (mean±SD, n = 4). (**B–E**) Immunohistochemistry stainings with MSC markers CD105, CD90, Bra and Snail1. (**F**) RT-PCR analysis displays presence of characteristic MSC markers CD90, ITGA6, GFRa1, CD73, CD105, ALCAMm CD44, whereas CD34 as marker for hematopoietic progenitors is absent (housekeeper RPS29). Scale bars are 50 µm.

Primary culture obtained from amnion membrane displayed high proliferation capacities until passage 26 (data not shown). Flow cytometry analysis demonstrated 87.56±4.92% of the cells were positive for MSC maker CD105 (ENG) and 85.00±4.28% – for CD73 (NT5E, Figure 2A). Immunohistochemistry showed positive brown to black reporter color for CD90 (THY-1), CD105 (ENG), Brachyury (Bra) and Snail-1 (Figure 2B–E). MSC characteristic genes like THY-1, ITGA6, GFRa1, CD73, CD105, ALCAM and CD44 were detected also in RT-PCR, whereas hematopoietic marker CD34 was absent (Figure 2F).

### Optimization of cell encapsulation

#### Effect of cell encapsulation on bead diameter

**Figure 3:**
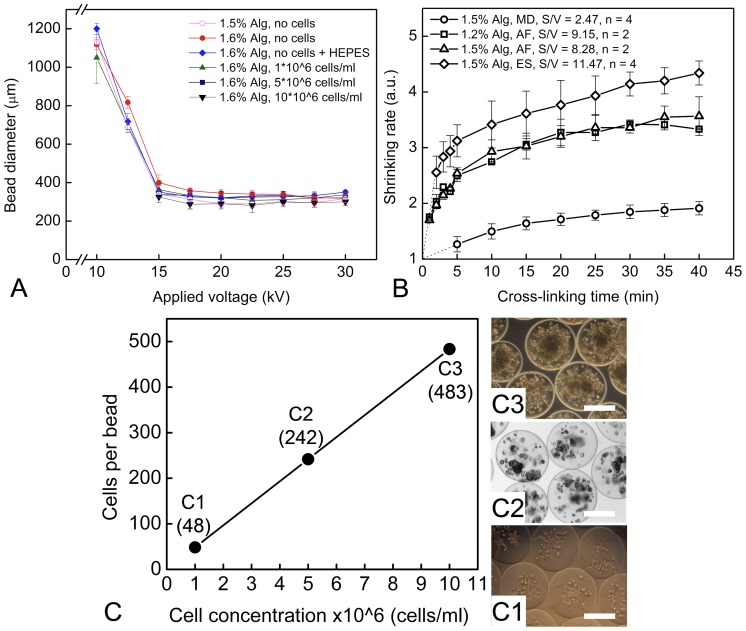
Optimization of cell concentration and cell number. (**A**) Effect of applied voltage, initial cell concentration and alginate-cell mixing procedure on diameter of alginate beads (mean±SD). (**B**) Shrinking of alginate in presence of 100 mM CaCl_2_ depending on gelling time for different initial surface-to-volume ratio (S/V) and (**C**) calculation of encapsulation efficiency of MSCs in alginate beads. Shrinking rate was analyzed based on decrease in volume during gelling caused by water release for: MD – manual dropping of alginate solution, AF – using air-flow method and ES – using electro-spraying; dotted lines show the tendency of shrinking outside the studied cross-linking time. (**C**) Effect of initial cell concentration on number of encapsulated cells and respective photographs of alginate encapsulated MSCs at initial concentration of 1×10^6^ (**C1**), 5×10^6^ (**C2**) and 10×10^6^ (**C3**) cells/ml. The next process parameters were kept constant: both for AF and ES – spraying distance 10 cm, concentration of gelling solution 100 mM CaCl_2_, alginate flow rate 10 ml/h, and alginate concentration from 1.2% to 1.6% (w/v); for AF – air flow 150 l/h; for ES – applied voltage 20 kV. The data are presented as a mean±SD (n = 3, N = 10). Scale bars are 200 µm.

For transplantation and pre-clinical studies, the size of alginate beads is one of the main factors, limiting diffusion of nutrients to encapsulated cells and vice versa the release of cell produced substances. The initial concentration of cells and alginate-cell mixing procedure may affect the resulting bead diameter and the performance of alginate beads after transplantation. In order to investigate these parameters, we encapsulated MSCs into 1.6% alginate with three different cell concentrations and under the range of voltage from 10 to 30 kV (Figure 3A). At the region below 10 kV, the strength of electric field was much lower than the surface tension, resulting in dropping mode and a bead size inapplicable for cell culture (>1 mm). At non stable region (10–15 kV), the slight increase in applied voltage had a pronounced effect on bead diameter, allowing to generate beads sized from 1,000 µm to less then 300 µm, respectively (dripping mode). Further increase in voltage did not significantly alter bead diameter (jetting mode). Such behavior has previously been reported, however, without encapsulated cells [27], [28]. In addition, concentration of 1.5% alginate was simulated by diluting appropriate volumes of 1.6% alginate with HEPES solution. Interestingly, an increase of cell concentration from 1×10^6^ to 10×10^6^ cells/ml of initial alginate did not have noticeable influence on bead diameter (Figure 3A), indicating that large number of cells can be encapsulated without altering the size of alginate beads.

#### Encapsulation efficiency

It is especially important in therapies with a cell-based drug delivery system to precisely calculate the amount of encapsulated cells in alginate beads. We investigated the efficiency of encapsulation by analyzing the shrinking rate of alginate during cross-linking. The shrinking rate of alginate (1.5% and 1.2%, w/v) in cross-linking solution without encapsulated cells depending on initial surface-to-volume ratio at defined time intervals and for a maximum of 40 min is shown in Figure 3B. Interestingly, the alginate beads possessing higher S/V ratio showed very fast and uncontrollable shrinking over the first 5 min of gelling, whereas those obtained by MD were shrinking stable and slow (Figure 3B, AF-ES *vs.* MD). In opposite, alginate drops generated via ES method and possessing the highest value of S/V ratio (11.47 mm^−1^) showed extremely fast shrinking during the first 5 min of cross-linking.

Calculating the number of cells to be entrapped in one bead (Figure 3C) with equation (2), the alginate beads contained 1×10^6^, 5×10^6^ and 10×10^6^ cells/ml and showed virtually homogeneous distribution of cells throughout the bead (Figure 3C, C1–C3). The cell concentration barely affected the size of alginate bead for a wide range of applied high voltage (Figure 3A). The average diameter of alginate beads was equal to 300 µm for each cell concentration. In exact numbers, we could show that it is possible to entrap 48 to 483 cells in an alginate bead for 1×10^6^ and 10×10^6^ cells/ml, respectively.

### Viability after encapsulation

**Figure 4:**
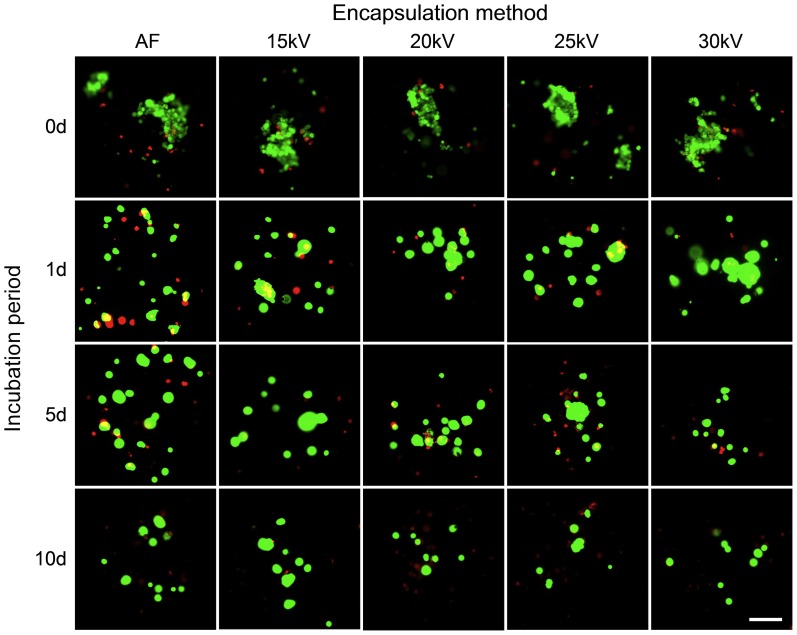
Viability of encapsulated MSCs *vs.* applied voltage and incubation time. Live-dead staining with CalceinAM (2 µM) and Ethidium Homodymer (4 µM). Note: beads obtained under 15 kV and using air flow (AF) had slightly bigger diameter as compared to the others. Viable cells stained in green, cells with massive membrane damages – in red. No visible difference in viability of encapsulated cells depending on applied voltage can be observed. The next process parameters were kept constant: both for AF and electro-spraying – spraying distance 10 cm, concentration of gelling solution 100 mM CaCl_2_, alginate flow rate 10 ml/h, and alginate concentration 1.6% (w/v); for AF – air flow 150 l/h. Scale bar is 100 µm.

In order to evaluate if the high voltage of the ES method affects the viability of cells especially by membrane damages, we utilized the trypan blue assay as a solid marker for membrane integrity of cells; however, the release of cells from the beads by immersing in sodium citrate and additional centrifugation may also lead to cell death by apoptosis. To reveal this effect, we stained encapsulated MSCs additionally at day 0, 1, 5, 10 with CalceinAM/EthD. We observed no noticeable effect of high voltage on viability of cells both post-encapsulation and incubation of MSCs inside alginate beads for 10 days as compared to AF (Figure 4, AF-30 kV). However, we noticed the negative effect of incubation time on the viability of encapsulated cells as compared to shorter incubation periods (Figure 4, day 10 *vs.* day 1). Despite this, most of cells were still viable at day 10. It should be noted that the effect of high-voltage on viability of encapsulated cells cannot be properly evaluated, since the beads obtained under 20, 25 and 30 kV had a smaller diameter as compared to AF and 15 kV. Thus, fewer viable cells were detected.

### Cell proliferation

#### Proliferation of encapsulated MSCs

**Figure 5:**
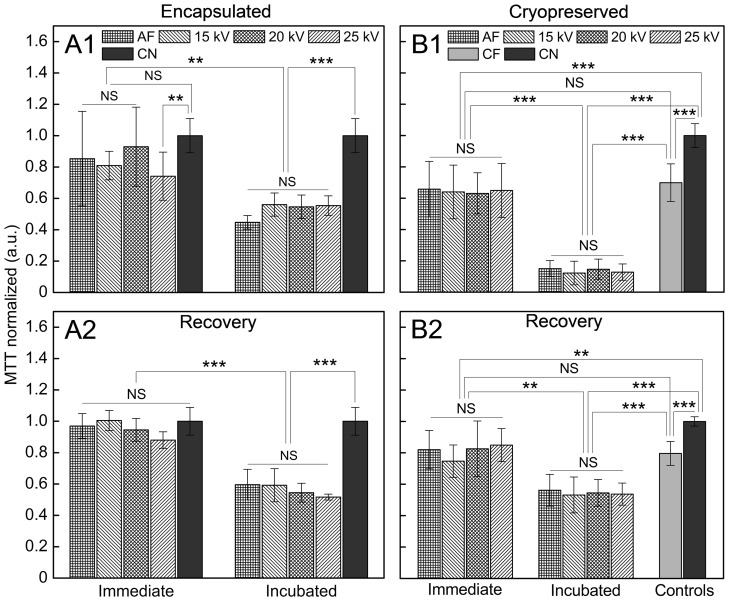
Effect of applied encapsulation methods and incubation on proliferation of MSCs post-encapsulation and cryopreservation. Proliferation of MSCs: (**A1)** after encapsulation in alginate; (**A2**) after recovery post-encapsulation; (**B1**) cryopreserved in alginate; (**B2**) after recovery post-thawing. “Recovery” – incubation of cells in culture (37°C, 5% CO_2_ in a humidified incubator) for 5 days after encapsulation and/or cryopreservation; “Immediate” – immediately after encapsulation; “Incubated” – after 24 h of incubation inside alginate beads in culture; AF – air flow; CF – frozen not encapsulated cells; CN – not encapsulated and not frozen MSCs. The next process parameters were kept constant during encapsulation: both for AF and electro-spraying – spraying distance 10 cm, concentration of gelling solution 100 mM CaCl_2_, alginate flow rate 10 ml/h, and alginate concentration 1.6% (w/v); for AF – air flow 150 l/h. In B1–B2 freezing parameters included cooling rate 1 K/min down to −80°C and freezing medium with 10% DMSO and 10% FBS. The data are shown as a mean±SD (n = 3, N = 5). One-way ANOVA: NS – not significant (p = 0.05); *, **, *** - significantly different with p<0.05, p<0.01, p<0.001, respectively.

We evaluated the effect of high voltage and incubation time of bead-enclosed cells on proliferation after encapsulation (Figure 5A1) and after recovery (Figure 5A2). Here, the encapsulation procedure and applied high voltage does not significantly affect the viability of encapsulated MSCs, as indicated by similar proliferation efficiency and metabolic activity as controls. Moreover, MSCs derived from alginate beads immediately after encapsulation recovered with the same rate as controls, but proliferation decreased significantly after 24 h of incubation. The recovery of MSCs post-encapsulation was dependent on pre-incubation time; such non-incubated cells (seeded for proliferation assay immediately after encapsulation) recovered at the same rate as a native control (Figure 5A2, “Immediate”). However, MSCs incubated in beads for 24 h showed a significantly lower recovery rate (Figure 5A2, “Incubated”).

#### Proliferation of encapsulated MSCs after cryopreservation

For long-term storage it is necessary to provide alginate encapsulated cells with high viability and proliferation after cryopreservation. The proliferation of frozen cells inside alginate structures incubated for 24 h (Figure 5B1, “Incubated”) was significantly lower in comparison with native control (Figure 5B1, “CN”). Interestingly, MSCs frozen inside alginate beads immediately after encapsulation revealed significantly higher proliferation rate as compared to the cells incubated for 24 h inside beads; whereas the proliferation rate was slightly lower for encapsulated and immediately frozen cells as compared to frozen control cells (Figure 5B1, “CF”). The recovery of MSCs post-cryopreservation was also affected by incubation time (Figure 5B2). In addition, MSCs frozen inside alginate beads possessed higher recovery rate as compared to frozen control cells and the same rate as fresh control cells (Figure 5B2, “CF”–“CN”). Application of high voltage to generate cell-containing alginate beads did not alter the metabolic activity of cells after thawing, as for “Immediate” and “Incubated” groups we did not observe the negative effect of high voltage on MSCs as compared to AF encapsulated ones (Figure 5B1–B2, 15–25 kV *vs.* AF).

In summary, the cryopreservation procedures reduced the number of cells with intact membrane. We showed that 62±5% of the cells were viable after thawing of cryopreserved MSCs directly after encapsulation, as compared to initially encapsulated ones (n = 3, N = 5). On the other hand, only 30±7% of cells were alive after applying pre-freeze incubation of MSCs inside alginate beads in culture for 24 h. We further detected the number of not encapsulated frozen MSCs of 76±3% after thawing, as compared to initially frozen cells. The number of cells recovered after thawing increased in 4.9 and 2.9 times after 5 days for immediately frozen and incubated for 24 h before cryopreservation (as compared to the number of cells seeded for recovery assay), respectively. According to our data, the number of not encapsulated frozen MSCs and fresh control increased in 4.6 and 4.8 times, respectively.

#### Proliferation of MSCs cultured inside beads for 7 days

**Figure 6:**
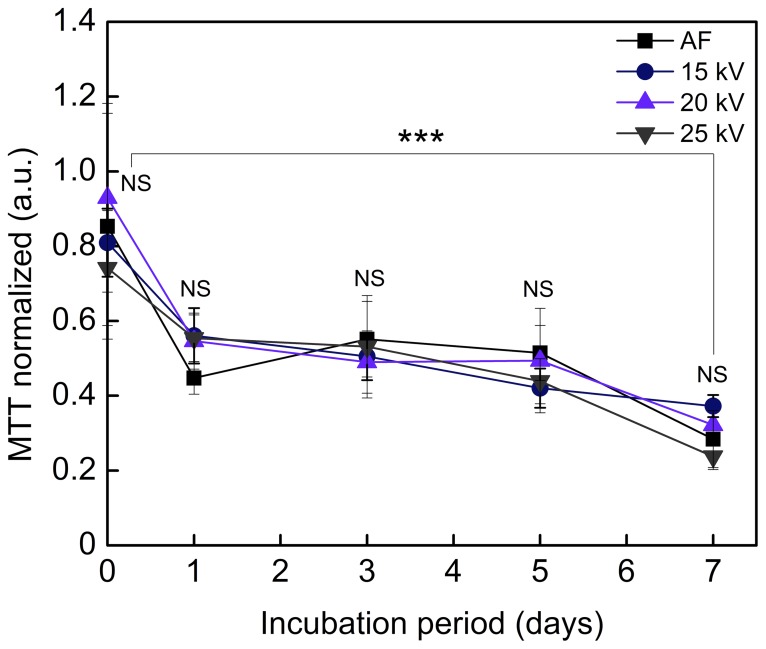
Effect of encapsulation method and period of incubation of MSCs inside beads on proliferation of cells. MSCs were encapsulated in alginate beads using air flow (AF) and high voltage (15, 20, 25 kV) and incubated for 0, 1, 3, 5 and 7 days inside beads in culture (37°C, 5% CO_2_ in a humidified incubator). Culture medium was replaced each second day to a fresh one. The next process parameters were kept constant: both for AF and electro-spraying – spraying distance 10 cm, concentration of gelling solution 100 mM CaCl_2_, alginate flow rate 10 ml/h, and alginate concentration 1.6% (w/v); for AF – air flow 150 l/h. The data are shown as a mean±SD (n = 2, N = 5). One-way ANOVA: NS – not significant (p = 0.05); *** – significantly different (p<0.001).

During evaluation of the effect of high voltage on proliferation of cells as compared to the AF method for a period of 7 days, we found that the viability and proliferation of encapsulated cells was decreasing during the incubation which resulted in significant decrease at day 7 independent from encapsulation method (Figure 6, day 7 *vs.* day 0). The cells seeded for proliferation immediately after encapsulation at 20 and 25 kV of high voltage showed slightly lower proliferation as compared to AF encapsulated MSCs. However, further incubation did not reveal significantly different effect of high voltage on the viability and proliferation of cells post-incubation as compared to AF method. In summary, the encapsulated cells proliferated slower as compared to non-encapsulated control cells.

### Differentiation capacity

**Figure 7:**
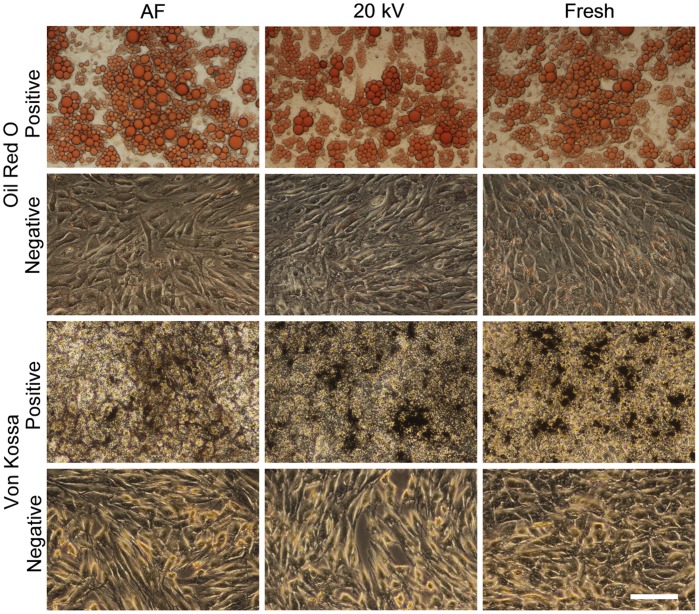
Differentiation capacity of encapsulated MSCs. MSCs were differentiated into adipogenic (Oil Red O) and osteogenic direction (Von Kossa) after encapsulation using air flow (AF) and high voltage (20 kV) followed by dilution of alginate with 55 mM sodium citrate for 5 min, or as not encapsulated MSCs. Cells expanded in regular culture medium represent the negative control (37°C, 5% CO_2_ in a humidified incubator) and stained the same as positive cells of respective lineage. The next process parameters were kept constant: both for AF and electro-spraying (ES) – spraying distance 10 cm, concentration of gelling solution 100 mM CaCl_2_, alginate flow rate 10 ml/h, and alginate concentration 1.6% (w/v); for AF – air flow 150 l/h; for ES – applied voltage 20 kV. Scale bar is 100 µm.

We studied the effect of high voltage on the capacity of encapsulated MSCs to differentiate into adipogenic and osteogenic lineages. MSCs were able to form adipocytes (Oil Red O staining) and osteocytes (Von Kossa staining) post-encapsulation (Figure 7). We detected no visible difference in the plasticity for MSCs encapsulated in alginate using high voltage (20 kV) and AF method to form lipid vacuoles (Figure 7, Oil Red O/Positive) and mineralize calcium phosphate (Figure 7, Von Kossa/Positive). Non-encapsulated MSCs also showed the capacity to differentiate into adipocytes and osteocytes (Figure 7, Fresh), whereas untreated control cells without differentiation media did not display vacuoles or mineralization.

## Discussion

High voltage has been widely used in bioengineering and regenerative medicine. Such applications include a) electrophoresis to extract DNA and proteins and b) electroporation to permeabilize the cell membrane to deliver genes and liposome into the cells and tissues to treat rare diseases and store rare cell types for long term [29]–[31]. In these approaches, the strength of electric field ranges from 1–10 V/cm of constant voltage for gel electrophoresis up to 100 kV/cm of pulse high voltage (the pulse duration can vary depending on application) for conventional electroporation (the pulse duration is in microsecond range) [32] and supra-electroporation (nanosecond pulsed electric field) causing high density nanopore formation in all cell membranes [33]. In our experiments we generated small-sized alginate beads with encapsulated MSCs applying the strength of electric field ranging from 1 kV/cm to 3 kV/cm. Comparing the high voltage method with the commonly used AF, we could show that the range of applied high voltage from 15 to 25 kV did not alter the viability, proliferation and differentiation capacity of MSCs after encapsulation in alginate. Although high voltage showed a significant effect on the proliferation rate of MSCs immediately after encapsulation depending on the method utilized (Figure 5A1, 25 kV *vs.* “CN”), there was no significant difference observed for 7 days in culture (Figure 6, “NS”).

The findings mentioned above correlate with our recent data on cell counts in studies after encapsulation and cryopreservation [34]. MSCs encapsulated in alginate beads may require longer periods of time to recover post-encapsulation; maybe due to limited diffusion of serum molecules through the negatively charged alginate porous structure - a problem that might get solved by applying higher FBS concentrations. It has previously been shown that an application of 3 kV/cm did not alter the viability of cells [35], [36]. In addition, the high voltage encapsulation of living cells supports cells with a mild environment during encapsulation that has also been suggested, but for impulse voltage [37].

Encapsulation of cells in alginate has been known since late 1960s and has been widely applied in mice, rats and rabbits as preclinical models [8]. However, the genotype and phenotype of these animals is very different compared to the human, especially in the field of stem cells research with an application of very experimental cell types like ESCs and iPS cells with very early features of cellular maturity. In the human, multipotent stromal cells are to date the only cell type already utilized in clinical trials [38]. This made them ideal assets in our experiments for extraction, cultivation, encapsulation and differentiation. True multipotent stromal cells with high “stemness” from the placental amnion of the common marmoset monkey can be natively obtained for personalized therapies and possible option of transplantation in our non-human primate model without ethical concerns or clinical safety problems.

The proliferation of encapsulated MSCs reduced after 7 days in culture as compared to not encapsulated control cells. It may be related to the restriction of space as well as the effects of hampered diffusion of nutrients and FBS to encapsulated cells, especially to centrally positioned ones, as it has been reported previously [39]. In addition, the negative effect of washing steps and liquefying procedures (release of cells from alginate beads) can not be excluded. One of the possible solutions could be an increase of concentration of FBS, along with a decrease in bead diameter, which could improve heat and mass transfer through the bead. A similar solution has also been suggested, showing that alginate has to be modified to allow attachment and proliferation of adherent mammalian cell types [40]–[42]. This could support our observation on a decrease of the viability and metabolic activity of encapsulated cells over the incubation period. On the other hand, the metabolic activity of encapsulated adherent mammalian cell types may be improved by modifying the initial alginate with RGD peptides [10]. These modifications have been shown to improve survival of cells after cryopreservation as compared to cells encapsulated in unmodified alginate. In this work, we showed that non-human primate MSCs encapsulated using high voltage can be readily stored at low temperatures for long term without altering the metabolic activity of cells after thawing, as compared to not encapsulated MSCs. In addition, by studying the shrinking rate of alginate we were also able to quantify the number of cells to be entrapped in one bead for the respective S/V ratio of 11.47 mm^−1^.

To our knowledge, there is to date no single study with a complete and comparative characterization of amnion derived encapsulated and cryopreserved MSCs including optimization of cell number, evaluation of metabolic activity of cells after encapsulation and cryopreservation and evaluation of the effect of encapsulation method on the differentiation potential of alginate encapsulated MSCs.

## Conclusions

The MSCs derived from a marmoset amnion proved to be true multipotent stromal cells with “stemness” and plasticity. They can be encapsulated in unmodified alginate beads at high concentration using high-voltage encapsulation with narrow size distribution and preserving the viability and proliferation of cells after encapsulation. In particular, optimization of process parameters of the ES method revealed only a slight influence of cell number on bead size. This indicates the possibility for large-scale generation of encapsulated cells that has previously been shown to be the critical point in translation of encapsulation technology to clinical application requiring high cell numbers [43]. The cells encapsulated using the high voltage method proliferated at the same rate as non-encapsulated cells and the cells encapsulated utilizing the traditional AF method. Incubation of MSCs in alginate in culture showed a significant decrease in proliferation 5 days after encapsulation, but MSCs were still viable at day 10. In addition, we showed that cryopreservation of MSCs inside alginate beads should be performed immediately after encapsulation to ensure high proliferation rates of cells after thawing. Further work should be aimed at establishing of alternative cryopreservation protocols for freezing of encapsulated MSCs with a control over the temperature of ice formation, which could improve the cryopreservation outcome. This work may give a background to further develop a preclinical therapy with high voltage alginate encapsulated MSCs in our non-human primate model towards human application.

## Supporting Information

Figure S1 **Thawing rate of alginate encapsulated MSCs.** DMSO was added to the sample at 4°C at a rate of 0.5 ml/min (drop-wise). The thermocouple was frozen at the center of a cryovial so that it was positioned at the point which separates equal volumes of freezing medium containing alginate encapsulated cells. The frozen sample was removed from −150°C and thawed immediately in a water bath at 37°C with gentle shaking until a small ice crystal was visible. The temperature increase was monitored each 3 seconds. Each point represents the results of measuring the thawing rate of 3 frozen samples.
(TIF)

Table S1 **Oligonucleotides and antibodies utilized for MSCs characterization.**
(DOC)
