# Supplementary figures and images for: Encapsulating Non-Human Primate Multipotent Stromal Cells in Alginate via High Voltage for Cell-Based Therapies and Cryopreservation
Source: PLoS One. 2014 Sep 26;9(9):e107911. doi: 10.1371/journal.pone.0107911 (PMC4178041; doi:10.1371/journal.pone.0107911)

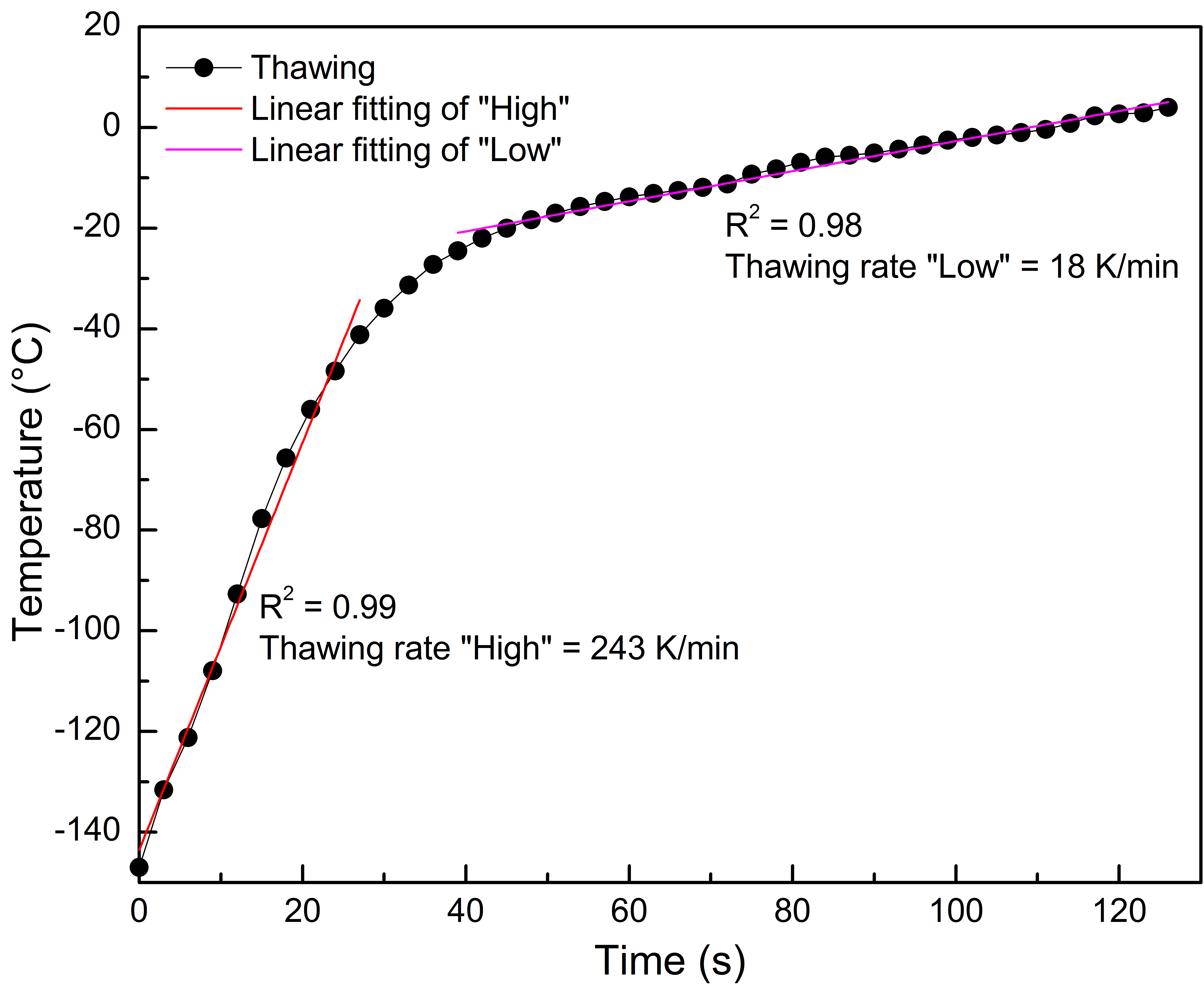

Supplement: Figure S1 — Thawing rate of alginate encapsulated MSCs. DMSO was added to the sample at 4°C at a rate of 0.5 ml/min (drop-wise). The thermocouple was frozen at the center of a cryovial so that it was positioned at the point which separates equal volumes of freezing medium containing alginate encapsulated cells. The frozen sample was removed from −150°C and thawed immediately in a water bath at 37°C with gentle shaking until a small ice crystal was visible. The temperature increase was monitored each 3 seconds. Each point represents the results of measuring the thawing rate of 3 frozen samples. (TIF) [file pone.0107911.s001.tif]
