# Supplementary material for: Encapsulating Non-Human Primate Multipotent Stromal Cells in Alginate via High Voltage for Cell-Based Therapies and Cryopreservation
Source: PLoS One. 2014 Sep 26;9(9):e107911. doi: 10.1371/journal.pone.0107911 (PMC4178041; doi:10.1371/journal.pone.0107911)
Supplement: Table S1 — Oligonucleotides and antibodies utilized for MSCs characterization. (DOC) [file pone.0107911.s002.doc]

**Table S1.** Oligonucleotides and antibodies utilized for MSCs characterization

| **Gene** | **(Sequence 5’→3’)** | **Annealing temperature, °C** | **Fragment** | **Reference** |
| --- | --- | --- | --- | --- |
| RPS29 | CGAAAATTCGGCCAGGGTTC  TGCCCCGGATAATCCTCTGA | 60 | 169 bp | ENSG00000213741 |
| THY1 (CD90) | TCCCAGAACGTCACTGTGCT  AGGGATAT AAATCCGTGGC | 60 | 134 bp | ENSCJAT00000027416 |
| ITGA6 (CD49) | AGAGAGCCAACAGAACGGGA  CATCCACTGATCTTCCTTGC | 60 | 122 bp | ENSG00000091409 |
| GFRA1 | TCAGTGCCTGAAGGAGCAGA  AGAGCGACTTCTGCTTCAGG | 60 | 152 bp | ENST00000334304 |
| CD73 | ACTCATCGCGCAGAAAGTGA  AACCTTCCGCCCATCTTCAG | 60 | 145 bp | XM_003732703 |
| CD105 | CTCTCCAGGCATCCAAGCAA  GGATGGCAGCTGTGTGGTAT | 60 | 179 bp | XM_002743382 |
| ALCAM (CD166) | ACGTGTTTGAGGCACCTACA  AGCTGCTCTGTTTCGAGAAA | 60 | 94 bp | ENSG00000170017 |
| CD44 | TGGCCTTGGCTTTGATTCTT  AGCTTTTTCTTCTGCCCACA | 60 | 73 bp | ENSG00000026508 |
| CD34 | CGCTCTGCTTGCTGAGTTTG  TGAGACACAGGGTACTGGCT | 60 | 178 bp | NM_001267735 |
| **Antibody** | **Company** |  | **Cat No.** | **Dilution** |
| Brachyury | Abcam | Rabbit Anti-Human | 20680 | 1:100 |
| CD90 | Abcam | Mouse Anti-Rat | 225 | 1:100 |
| CD105 | Dianova | Mouse Anti-Human | 07243 | 1:100 |
| CD73 | Abcam | Mouse Anti-Human | 54217 | 1:250 |
| Snail1 | Santa Cruz | Rabbit Anti-Human | 28199 | 1:100 |
| Alexa Fluor 488 | Life Technologies | Donkey Anti-Mouse | A21202 | 1:400 |
